# Advances in Entomopathogen Isolation: A Case of Bacteria and Fungi

**DOI:** 10.3390/microorganisms9010016

**Published:** 2020-12-23

**Authors:** Lav Sharma, Nitin Bohra, Vishnu D. Rajput, Francisco Roberto Quiroz-Figueroa, Rupesh Kumar Singh, Guilhermina Marques

**Affiliations:** 1 Centre for the Research and Technology of Agro-Environment and Biological Sciences, Universidade de Trás-os-Montes e Alto Douro, Quinta de Prados, 5000-801 Vila Real, Portugal; gmarques@utad.pt; 2 Max Planck School Matter to Life, Max Planck Institute for Medical Research, Jahnstraße 29, 69120 Heidelberg, Germany; nitin.bohra@mtl.maxplanckschools.de; 3 Soil Science and Land Evaluation Department, Academy of Biology and Biotechnology, Southern Federal University, 344090 Rostov-on-Don, Russia; rajput.vishnu@gmail.com; 4 Instituto Politécnico Nacional, Centro Interdisciplinario de Investigación para el Desarrollo Integral Regional Unidad Sinaloa (CIIDIR-IPN Unidad Sinaloa), Laboratorio de Fitomejoramiento Molecular, Blvd. Juan de Dios Bátiz Paredes no. 250, Col. San Joachín, C.P., Guasave 81101, Mexico; labfitomol@hotmail.com; 5 Centro de Química de Vila Real, Universidade de Trás-os-Montes e Alto Douro, Quinta de Prados, 5000-801 Vila Real, Portugal; rupesh@utad.pt

**Keywords:** *Beauveria*, *Metarhizium*, Hypocreales, *Bacillus thuringiensis*, *Serratia*

## Abstract

Entomopathogenic bacteria and fungi are quite frequently found in soils and insect cadavers. The first step in utilizing these microbes as biopesticides is to isolate them, and several culture media and insect baiting procedures have been tested in this direction. In this work, the authors review the current techniques that have been developed so far, in the last five decades, and display brief protocols which can be adopted for the isolations of these entomopathogens. Among bacteria, this review focuses on *Serratia* spp. and bacteria from the class Bacilli. Among fungi, the review focuses those from the order Hypocreales, for example, genera *Beauveria*, *Clonostachys*, *Lecanicillium*, *Metarhizium*, and *Purpureocillium*. The authors chose these groups of entomopathogenic bacteria and fungi based on their importance in the microbial biopesticide market.

## 1. Introduction

The global biopesticide market is expected to reach around USD 7.7 billion with a compound annual growth rate of 14.1% [1]. It is also estimated that microbial biopesticides will account for 3% of the total pesticide market [2]. The shift toward microbial biopesticides is increasing as European legislation is continuously pressing to minimize the residue levels of synthetic chemical pesticides. Moreover, forthcoming directive (EC 91/414) demands a ban of chemical pesticides that are deemed to be the disruptors of human endocrine system. Microbial biocontrol agents are the new hope in this direction, and governments and scientists in Europe have simplified the European microbial pesticide registration procedures outlined in the Regulation of Biological Control Agents (REBECA), with an objective to facilitate the development of microbial biocontrol agents [3].

Entomopathogenic bacteria (EPB) and entomopathogenic fungi (EPF) are the natural enemies of insect-pests. Hence, their importance in agriculture is quite high [4,5,6,7,8]. The majority of the EPB belong to a few bacterial families, such as Bacillaceae, Enterobacteriaceae, Micrococcaceae, Pseudomonadaceae, and Streptococcaceae. *Bacillus thuringiensis* (*Bt*) is arguably the most widely studied and used bacterial entomopathogen [9]. At present, there are over 40 *Bt* products for insect biological control, which account for 1% of the total global insecticide market and approximately a market of USD 210 million per annum [3,10,11]. Other bacterial biopesticides account for approximately USD 50 million per annum. A list of commercial EPB and their target insect groups is presented in the Table 1.

Similarly, over 170 biopesticides based on fungi have been developed since 1960, and 75% are either still in use or have been registered [10,11]. This accounts for at least USD 77 million annually [3,10,11]. Their popularity can be attributed to the fact that EPF pose lesser risks for nontarget arthropods, such as bees, predatory beetles, and parasitic wasps. Hypocrealean fungi such as *Beauveria*, *Metarhizium*, *Cordyceps*, and *Lecanicillium* are some of the well-known fungal entomopathogens [7]. A list of commercially available EPF along with their target insect groups is presented in the Table 2.

Some culture-independent techniques have also been employed for the detection and quantification of EPB and EPF, for example, in the case of EPB, amplifying the region of 16S ribosomal DNA from the bacteria *Pseudomonas entomophila* by employing a duplex polymerase chain reaction (PCR) and further validating the method in *P. entomophila*-infected *Drosophila melanogaster* Meigen (Diptera: Drosophilidae) [12] or designing primers for *Bacillus thuringiensis* serovar *israelensis* and testing them using soil samples [13]. Similarly, for EPF, quantitative PCR approaches have been employed, such as amplifying the ITS region of *Metarhizium* from soil samples [14]; employing validated simple sequence repeats’ primers for *Beauveria* detection [15]; amplifying minute quantities of DNA of *Beauveria bassiana* in host plant using a two-step nested PCR with the primer pairs, ITS1F/ITS4, and BB.fw/BB.rv [16]; or a two step-nested PCR method to detect *Beauveria* samples in rhizosphere by amplifying translation elongation factor 1-aplha (*tef1*-*α*) gene [17]. However, such culture-independent studies are out of the scope of this review. In this review, the authors describe recent laboratory techniques that are based on insect baiting and culture-based methodologies to eventually isolate EPB and EPF from soils or from insect cadavers collected from the fields. Nonetheless, EPB and EPF are quite diverse, hence this review focuses on the most commonly occurring EPB and EPF.

## 2. Isolation of Entomopathogenic Bacteria

Entomopathogenic bacteria are commonly found in soils. Hence, isolating insect-pathogenic strains is quite important. Different bacterial groups, such as symbionts of entomopathogenic nematode (EPN) *Heterorhabditis* spp. and *Steinernema* spp., i.e., *Photorhabdus* spp. and *Xenorhabdus* spp., and others, such as *Yersinia entomophaga*, *Pseudomonas entomophila*, and *Chromobacterium* spp., exhibit entomopathogenicity [18].

Entomopathogenic nematode symbiotic bacteria are isolated by dropping an insect’s hemolymph onto a nutrient bromothymol blue (0.0025% (*w/v*)) triphenyltetrazolium chloride (0.004% (*w/v*)) agar (NBTA) and incubating the streaked plate at 25 °C, and continuously subculturing until the uniform colonies are obtained [19]. *Yersinia entomophaga* is isolated by culturing the hemolymph of diseased larvae of New Zealand grass grub, *Costelytra zealandica* White (Coleoptera: Scarabaeidae), onto Luria-Bertani (LB) agar, followed by growth on Caprylate-thallous agar (CTA) (Appendix A, Medium 1) and Deoxyribonuclease (DNase)-Toluidine Blue agar (Appendix A, Medium 2), and no hemolysis on Columbia horse blood agar (Columbia agar + 5% horse blood) or Columbia sheep blood agar (Columbia agar + 5% sheep blood) [20]. Isolating *P. entomophila* is rather tricky as the bacterium needs to elicit the systemic expression of Diptericin, an antimicrobial peptide in *Drosophila*, after ingestion. However, the bacterial culture can be maintained on LB media [21]. Bacterial isolates from insects belonging to *Chromobacterium* exhibit violet pigment when cultured on L-agar [22]. However, EPB that are most commonly used as commercial biopesticides are further discussed in the review.

### 2.1. Milky Disease-Causing Paenibacillus spp.

*Paenibacillus popilliae* and *Paenibacillus lentimorbus* are obligate pathogens of scarabs (Coleoptera) as they require the host for the growth and sporulation. In soils, they are present as endospores. These bacteria can be isolated from the hemolymph, and the methodologies may vary depending on the bacterial species. The protocols listed below have been described by Stahly et al., and more details of these protocols have been reported by Koppenhöfer et al. [23,24,25].

(a) Disinfect the surface of the larvae of grubs (Coleoptera) with 0.5% (*v*/*v*) sodium hypochlorite (NaOCl).
(b) Pinch the cadaver using a sterilized needle and collect the emerging drops in sterilized water.
(c) Culture the dilutions of the drops on St. Julian medium (J-Medium) (Appendix A, Medium 1) [26], or Mueller-Hinton broth, yeast extract, potassium phosphate, glucose, and pyruvate (MYPGP) (Appendix A, Medium 2) agar [27].

Note: To enhance the germination of the vegetative cells, using 0.1% (*w*/*v*) tryptone solution is recommended during bacterial dilutions [26]. For spores, it is advisable to heat them for 15 min in a 1 M calcium chloride solution (pH 7.0) at 60 °C, and suspend them in the hemolymph of the cabbage looper *Trichoplusia ni* Hübner (Lepidoptera: Noctuidae) and in tyrosine at an alkaline pH. Another way to improve the germination is to heat the spores at 75 °C for 30 min and then apply pressure using a French press [28].

Alternatively, another method described by Milner [29] can be used, which utilizes the poor germination of *P. popilliae var. rhopaea*.

(a) Make soil suspensions by adding 2 g soil to 20 mL sterilized water.
(b) Make a germinating medium, i.e., 0.5% yeast extract and 0.1% glucose.
(c) Adjust the pH to 6.5.
(d) Add germinating medium into the soil suspension at 1:50 ratio.
(e) Apply series of heat shocks at 70 °C for 20 min after every hour, 7 times.
(f) Spread the aliquot on J-Medium and incubate for 7 h at 28 °C, anaerobically.

To save time and quantify spores, Stahly et al. [23] gave another methodology which capitalizes on *P. popilliae* resistance to vancomycin. In this method, soil suspensions are plated on MYPGP agar with 0.015% (*w*/*v*) vancomycin. Not all *P. popilliae* strains are vancomycin-resistant, hence this method should be used with caution. Moreover, fungal contamination can be avoided by adding cycloheximide 0.01% (*w*/*v*) and incubating for 3 weeks at 30 °C.

### 2.2. Amber Disease-Causing Serratia spp.

*Serratia* spp. are quite frequently isolated from soils, and some of them, being saprophytes, can also be isolated from insect cadavers. Therefore, to enhance the growth of insect pathogenic *Serratia* spp. such as *Serratia entomophila*, *Serratia proteamaculans*, and *Serratia marcescens*, a methodology based on a selective agar medium has been described by O’Callaghan and Jackson [30].

(a) Soil inoculums or hemolymph of the diseased larvae can be isolated on Caprylate-thallous agar (CTA) (Appendix A, Medium 3) [31].
(b) Culturing is done by pulling and separating the anterior end of the cadavers. The gut contents are then cultured on CTA plates.
(c) *Serratia marcescens* produces colonies which are red in color. Cream-colured bacterial colonies formed on CTA can then be transferred into different selective media for the identification of *Serratia* spp. [30].
(d) The production of a halo on a Deoxyribonuclease (DNase)-Toluidine Blue agar (Appendix A, Medium 4) when incubated at 30 °C for 24 h, indicates the presence of *Serratia* spp. [32]. Thereafter, the production of blue or green colonies on adonitol agar (Appendix A, Medium 5) confirms *S. proteamaculans*. The formation of yellow colonies on adonitol agar hints the presence of *S. entomophila*, which can be confirmed by the growth on itaconate agar (Appendix A, Medium 6) at 30 °C after 96 h [25]. Further molecular approaches targeting specific DNA regions can distinguish pathogenic strains from the non-pathogenic ones.

### 2.3. Other Bacteria from the Class Bacilli

**Figure 1:**
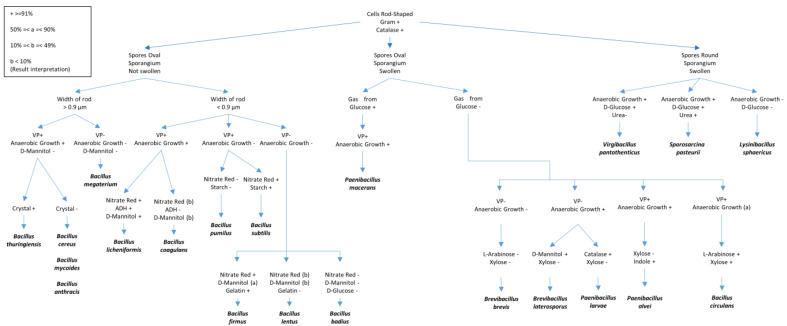
Different biochemical tests for the identification of Bacilli species. The figure was adapted and redrawn after modifications from T.W. Fisher and Garczynski [23]. Some details of the tests presented include VP (Voges–Proskauer test (Barritt’s method)), Gelatin (proteolysis of gelatin), ADH (presence of the amino acid arginine dihydrolase), Glucose (fermentation) and Mannitol (fermentation); Starch (hydrolysis), Nitrate (nitrate reduction to nitrite), and Urea (Urease test).

In general, bacterial species from the class Bacilli are commonly isolated from soils, insects, and water samples. Some species such as *Bt* produce heat-resistant endospores, which enhance the isolation of the bacterium of interest only. The common protocol for the isolations of Bacilli is as follows:

(a) Isolation can be done from soils (2–4 g in 10 mL sterilized water), insects (0.2–0.4 g/mL sterilized water), or water samples (after concentrating using 0.22 µm filter).
(b) Heat the samples in a water bath at 80 °C for 10 min to kill the vegetative cells.
(c) Perform serial dilutions, generally at 10^−2^ and 10^−3^, and culture the inoculums on Minimal Basal Salt (MBS) medium (Appendix A, Medium 7), as suggested by Kalfon et al. [33]. Continue subculturing until pure cultures are obtained.
(d) Perform bacterial identifications using different biochemical tests and 16S rDNA sequencing. Tests used to identify the bacteria within the class Bacilli are shown in the Figure 1, as described by T. W. Fisher and Garczynski [34].

## 3. Isolation of Entomopathogenic Fungi

Fungal entomopathogens can directly be isolated from insect cadavers in the case of visible mycosis [35]. Moreover, they can also be isolated from soils or phylloplane as they spend a considerable part of their life as saprophytes in soils or as plant endophytes. However, to our knowledge, their survival as soil saprophytes has not been proven yet [4,5,6,7,8,35,36]. In either case, the material can be cultured directly onto a medium selective for an EPF or the material can be baited with an infection-sensitive insect [37]. In case of the isolation of EPF as endophyte, proper disinfection of the material is needed. Nonetheless, different antibacterial and fungal saprophyte-inhibiting chemicals are added in the selective medium, as per the research interest. Here, different culture media used to isolate fungal entomopathogens, especially those belonging to the order Hypocreales are discussed.

### 3.1. Isolations from Naturally Mycosed Insect Cadavers

This method is applied to study the natural EPF infections in the fields as it relies on the collection of the dead insects from the fields. The protocol described below is similar to that employed by Sharma et al. [7].

(a) Insect cadavers are brought to the laboratory as separate entities in sterile tubes.
(b) Insects are observed under a stereomicroscope (40×) for probable mycosis.
(c) In case of a visible mycosis, the insects are surface sterilized using 70% ethanol or 1% NaOCl, for 3 min, followed by 3 distinct washes with 100 mL of sterilized water. Then, the sporulating EPF from the insect cadaver is plated directly.
(d) Cadavers are then cultured on a selective medium at 22 °C for up to 3 weeks, depending on the time taken by the fungi for germination and proliferation. In case of no germination, the cadavers can be homogenized and plated on the selective medium. Details of the different selective medium are provided later in the text.
(e) Obtained fungi are subcultured on potato dextrose agar (PDA) (Appendix A, Medium 8) or Sabouraud dextrose agar (SDA) (Appendix A, Medium 9) until pure culture is obtained.
(f) Fungi are identified by comparing morphological characteristics using light microscopy (400×), described in several fungal identification keys, such as Domsch et al. [38] and Humber [39].
(g) Molecular identifications can be done by extracting the DNA and performing PCR for the amplification and subsequent sequencing of the nuclear internal transcribed spacer (nrITS) region of the fungal nuclear ribosomal DNA, as described in Yurkov et al. [40].

Note: If the objective of the work is to study the diversity of the fungal entomopathogens, irrespective of the genus of interest, a few media can be used: (a) SDA with 0.2% yeast extract (*w/v*), i.e., SDAY further supplemented with 0.08% (*w/v*) streptomycin-sulphate and 0.03% (*w/v*) penicillin [41]; (b) SDA supplemented with 0.05% (*w/v*) streptomycin-sulphate and 0.025% (*w/v*) chloramphenicol [42]; (c) PDA supplemented with either 0.01% (*w/v*) streptomycin-sulphate and 0.005% (*w/v*) tetracycline [43], 0.01% (*w/v*) chloramphenicol [44,45], or 0.01% (*w/v*) penicillin, 0.02% (*w/v*) streptomycin-sulphate and 0.005% (*w/v*) tetracycline [46]; (d) oatmeal agar supplemented with 0.06% (*w/v*) cetyl trimethyl ammonium bromide and 0.05 % (*w/v*) chloramphenicol (OM-CTAB) (Appendix A, Medium 10) [47]; (e) Dichloran Rose Bengal chloramphenicol agar (DRBCA) [4,48] (Appendix A, Medium 11), or DRBCA supplemented with 0.05% (*w/v*) streptomycin-sulphate [37]. It is always advisable to use more than one selective medium pertaining to the susceptibility of a few EPF species to a particular concentration of the inhibitory chemical used.

### 3.2. Isolations from Soils

Isolations of fungal entomopathogens from soils can be done in 2 ways, i.e., either by culturing the soil inoculums or by employing bait insects. In any of the cases, after visible mycosis, the steps are similar to those described in Section 3.1. If the research objective is to isolate a particular EPF genus, then the relevant selective medium described below can be used. The details of the constituents of these selective media used for EPF isolation are given in Appendix A.

#### 3.2.1. Soil Suspension Culture

This method is generally used to isolate a particular EPF genus of interest using different concentrations of the soil inoculums. To ensure correct isolation, the isolated EPF should also be characterized morphologically and molecularly, as described in Section 3.1. Here the authors discuss various selective media used, especially those which are useful for the isolation of the hypocrealean fungi pertaining to their dominance in fungi-based microbial pesticide market. 

#### *Metarhizium* spp.

Isolating EPF has always been challenged by the contamination from saprophytic fungi. In this direction, Veen and Ferron [49] suggested using dodine (*N*-dodecylguanidine monoacetate) to inhibit the growth of saprophytes and developed Veen’s semi-selective medium to accomplish this (Appendix A, Medium 12). Later, Chase et al. [50] and Sneh [51] also used dodine in their studies. However, Liu et al. [52] reported that the higher quantities of dodine can be inhibitory to EPF and suggested using only 10 µg/mL dodine (Appendix A, Medium 12). Later, Rangel et al. [53] cautioned against the use of dodine and showed the even 0.006% (*w/v*) dodine in PDAY can completely inhibit *Metarhizium acridum*. This led to the development of CTC medium, which is made by the addition of 0.05% (*w/v*) chloramphenicol, 0.0001% (*w/v*) thiabendazole, and 0.025% (*w/v*) cycloheximide in PDAY [54] (Appendix A, Medium 13). However, a recent study by Hernández-Domínguez et al. [55] suggested the use of CTC medium, along with other dodine-containing mediums, for better *Metarhizium* recoveries. Posadas et al. [47] demonstrated that OM-CTAB is effective in isolating EPF while inhibiting saprophytes. Moreover, this negated the dependency on dodine, as it is not easily available in some countries.

#### *Beauveria* spp.

*Beauveria* spp., e.g., *Beauveria bassiana* sensu lato (s.l.) and *Beauveria pseudobassiana*, can be easily isolated using oatmeal dodine agar (ODA), as described by Chase et al. [50] (Appendix A, Medium 14). This medium has also been used in recent studies [56,57,58,59]. Another medium, i.e., Sabouraud-2-glucose agar (S2GA), was made by Strasser et al. [60] (Appendix A, Medium 15) for the isolation of *Beauveria brongniartii*, and was successfully used in studies concerning *B. brongniartii* [61,62,63]. However, many recent studies have used S2GA, with slight modifications, to isolate of *B. bassiana* s.l. [64,65]. A dodine-free alternative in isolating *B. bassiana* s.l. is OM-CTAB [47]. Moreover, Ramírez-Rodríguez and Sánchez-Peña [66] suggested using PDAY with CTAB (0.015% or 0.03% (*w/v*)) and any of the antibacterial compounds, i.e., dihydrostreptomycin, oxytetracycline, or doxycycline, to isolate *Beauveria* while inhibiting fungal saprophytes.

#### *Purpureocillium* spp.

*Purpureocillium* spp., i.e., *Purpureocillium lilacinum* and *Purpureocillium lavendulum*, can easily be isolated using an agar medium containing sodium chloride, benomyl, pentachloronitrobenzene, and Tergitol [67,68] (Appendix A, Medium 16).

#### *Lecanicillium* spp.

A *Lecanicillium*-selective medium (LSM) was developed by Kope et al. [69]. OM agar with 0.05% (*w/v*) chloramphenicol and 0.05% (*w/v*) CTAB can also be used, as described recently by Xie et al. [70] (Appendix A, Medium 17).

#### *Clonostachys* spp.

*Clonostachys* spp., e.g., *Clonostachys rosea* f. *rosea*, is reported entomopathogenic and can be isolated frequently from soils. Culture medium such as DRBCA is highly effective in isolating *Clonostachys* spp., at least in the case of the isolations from cadavers [7].

#### 3.2.2. Insect Baiting

This method is arguably the most commonly used method for EPF isolation, as the bait insect specifically selects entomopathogens from other saprobes in the soils [35,71,72], although surface sterilization of the insect cadavers is needed to avoid occasional contaminations by saprophytic fungi. 

#### *Galleria*-Bait Method or *Tenebrio*-Bait Method

The use of *Galleria mellonella* Linnaeus (Lepidoptera: Pyralidae) for isolating EPF from soil or the “*Galleria*-bait method” was first described by Zimmermann [73]. Since then, it has been used for EPF isolations in many studies [74,75,76,77,78,79,80,81,82,83,84,85,86,87,88,89,90,91]. *Tenebrio molitor* Linnaeus (*Coleoptera: Tenebrionidae*) has also been used as a bait insect in some studies [92,93,94]. Some previous studies have noticed that insect baiting is more sensitive in isolating EPF than culturing soil suspensions on selective medium [61,62,95,96]. Other studies have also used insect baiting along with soil suspension cultures [57,97,98,99,100]. Although insect baiting is a widely accepted method for EPF isolation, it should be used with caution as some lines of insect baits, such as the dark (melanic) morphs of *G. mellonella*, are more resistant to *B. bassiana* s.l.., and this trait has also been observed in *T. molitor* for *M. anisopliae* s.l. [101,102]. Similarly, immune-suppressed *G. mellonella* were found to be highly (~200 times) susceptible to EPF, which can lead to the isolation of a diverse set of EPF from soils, although saprophytic fungi may not induce any insect mortality [103].

#### *Galleria-Tenebrio*-Bait Method

**Figure 2:**
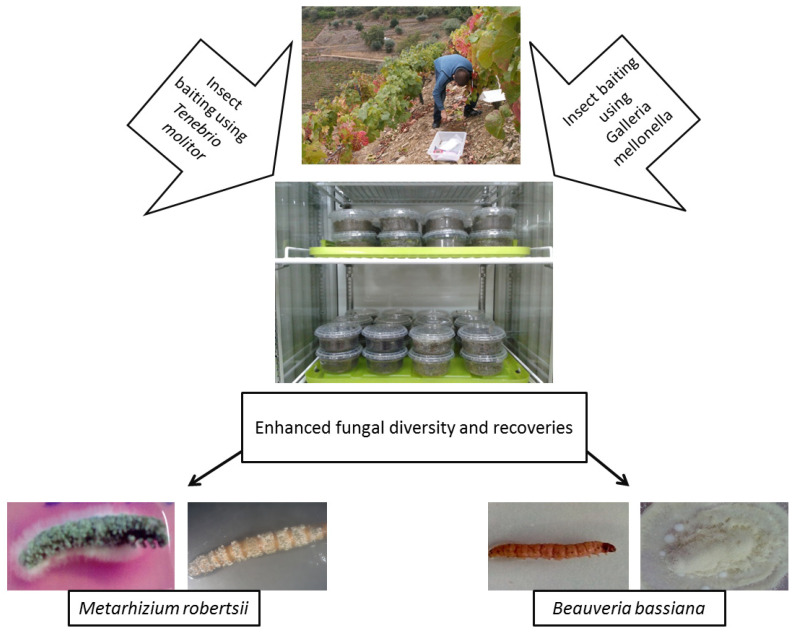
Isolation of entomopathogenic fungi from soils using the “*Galleria*-*Tenebrio*-bait method” The method has been described in detail by Sharma et al. [7].

As bait insects can be sensitive to infection by one particular EPF genus, some studies have used both *G. mellonella* and *T. molitor* to isolate EPF, either in part or throughout their whole experiment [7,104,105,106,107]. Recently, Sharma et al. [7] suggested using the “*Galleria*-*Tenebrio*-bait method” to avoid any underestimation of EPF abundance and diversity, as it was found that *G. mellonella* and *T. molitor* were significantly more sensitive toward the infections by *B. bassiana* s.l. and *M. robertsii,* respectively. This method is described in Figure 2.

#### Other Bait Insects

Several other bait insects have also been used along with either or both of the common bait insects described above. For example, Vänninen [104] used *Tribolium castaneum* Herbst (*Coleoptera: Tenebrionidae*) and *Acanthocinus aedilis* Linnaeus (*Coleoptera:* Cerambycidae), Klingen et al. [108] employed *Delia floralis* Fallén (Diptera: Anthomyiidae), Goble et al. [109] used *Ceratitis capitata* Wiedemann (Diptera: Tephritidae) and *Thaumatotibia leucotreta* Meyrick (Lepidoptera: Tortricidae), and Rudeen et al. [110] used *Diabrotica virgifera virgifera* LeConte (Coleoptera: Chrysomelidae).

### 3.3. Isolation from Phyllosphere

Some studies have also isolated EPF from the phylloplane and other parts of the plant phyllosphere, as these fungi can also be present as plant epiphytes or endophytes [41]. Meyling et al. suggested a leaf imprinting methodology where the leaf is cultured onto a selective agar medium [64]. Petri dishes with partitions are used and the upper (adaxial), and the lower (abaxial) surface of the leaf are pressed on the separate sides of the petri plate. Henceforth, the same leaf is put on a paper sheet and photocopied to estimate its surface area using image analysis software at a later stage. The petri plates are incubated in the dark at 23 °C to count fungal colony forming units (CFUs) [64]. Surface sterilization is quite important in isolating hypocrealean fungi as endophytes. This can be done by dipping the plant part in either 70% ethanol and/or 1–5% NaOCl for 3 min. In case of the leaves, the petiole can be first kept out of the sanitizer to avoid the chemical reaching inside the leaf, and then it can be cut to culture the sterilized part of the leaf on either of the selective mediums described above. It is always recommended to sanitize the intact plant part and then cut it into pieces for further culturing, as this avoids the sterilization of the endophytic fungi [111]. Different studies have isolated EPF from the phyllosphere, such as bark and branch samples [56,112] and leaves [59,113]. Nonetheless, Table 3 summarizes different studies performed to isolate EPF either using soil suspension on selective media and/or bait-insect(s), as these two methods were found to be the most common. 

### 3.4. Molecular Identifications of the Isolated Entomopathogenic Fungi

After obtaining a single spore fungal culture on a PDA or SDA (Appendix A; Medium 8 and/or 9), as described in the Section 3.1, the species can be resolved or identified by amplifying the regions of nuclear ribosomal DNA, such as *nrITS*, large (28S) subunit (*nrLSU*), or small (18S) subunit (*nrSSU*). Another, nuclear ribosomal DNA region, i.e., the intergenic spacer region between *nrSSU* and *nrLSU* or *IGS*, has also been used to understand *Beauveria* and *Metarhizium* speciation [113,114,115,116]. The resolution of the molecular identification can be increased by amplifying other nuclear DNA regions of interest, e.g., for Bloc for *Beauveria* [113,114,115] and the 5′ intron-containing region of translation elongation factor 1-alpha subunit (5′-*tef1α*) for *Metarhizium* [116,117]. Other nuclear DNA markers, such as the regions of the gene encoding for the largest subunit of RNA polymerase II (*rpb1*), the second largest subunit of RNA polymerase II (*rpb2*); β-tublin (*β-tub*), and the coding region of Tef1-α, can also be employed, in general, for any EPF [118,119].

Moreover, in the last decades, researchers have been constantly developing and validating the use of several microsatellite markers for the genotyping of *Beauveria* [93,115,120,121,122,123] and *Metarhizium* [124,125] isolates. For example, Oulevey et al. [125] described 18 small single repeats or microsatellite marker sets for *Metarhizium*, i.e., Ma145, Ma325, Ma307, Ma2049, Ma2054, Ma2055, Ma2056, Ma2057, Ma2060, Ma2063, Ma2069, Ma2070, Ma2077, Ma2089, Ma2283, Ma2287, Ma2292, and Ma2296. Similarly, Meyling et al. [93] and Goble et al. [123] validated the use of 17 to 18 microsatellite marker sets for *Beauveria*, i.e., Ba06, Ba08, and Ba12-Ba29. This methodology enables enhanced resolution among very closely related isolates which may otherwise be rendered as clones. Recently, Kepler and Rehner [119] developed primers for the amplification and sequencing of nuclear intergenic spacer markers for the resolution of *Metarhizium* isolates, i.e., BTIGS, MzFG543, MzFG546, MzIGS2, MzIGS3, MzIGS5, and MzIGS7, and Kepler et al. [99] successfully validated the use of MzIGS3 and MzFG543 on the *Metarhizium* isolated from agricultural soils.

## 4. Conclusions

Culture-based techniques are the classical approach for the quantification of microbial abundance and diversity. With the discoveries of entomopathogens, such approaches have been extended for these beneficial microbes. Moreover, techniques such as insect baiting also enhance their detection, even when the quantities are low. In the last few decades, the literature has highlighted the reproducibility of these methodologies [127]. With an increase in studies concerning the diversities of entomopathogens and with the advent of newer chemicals, more culture media will come into play. Simultaneously, to understand the abundance of entomopathogens in samples such as soils and plant tissues, culture-independent techniques such as metagenomics will also assist lab-based results.

## Figures and Tables

**Table 1:** Examples of common commercially available entomopathogenic bacteria (EPB) and their target insect groups.

Bacteria	Target Pest	Crops	PRODUCT (Company, Country)
*B. acillus thuringiensis subsp. kurstaki*	Lepidoptera	Row crops, forests, orchards, forests turfs	CRYMAX (Certis, USA)
			DELIVER (Certis, USA)
			JAVELIN WG (Certis, USA)
			COSTAR JARDIN; COSTAR WG (Mitsui AgriScience International NV, Belgium)
			LEPINOX PLUS (CBC, Europe)
			BACTOSPEINE JARDIN EC (Duphar BV, The Netherlands)
			DOLPHIN (Andermatt Biocontrol, Switzerland)
			BMP 123 (Becker, USA)
			DIPEL DF (Valent Biosciences, USA)
			LEAP (Valent Biosciences, USA)
			FORAY 48 B (Valent Biosciences, USA)
*B. thuringiensis subsp. aizawai*	Lepidoptera	Row crops, orchards	CRYMAX (Certis, USA)
			AGREE 50 WG (Certis, USA)
			XENTARI (Valent Biosciences, USA)
			FLORBAC (Bayer, Germany)
*B. thuringiensis subsp. tenebrionis*	Coleoptera: Chrysomelidae	Potatoes, tomatoes, eggplant, elm trees	TRIDENT (Certis USA)
			NOVODOR FC (Valent Biosciences, USA)
*B. thuringiensis subsp. israelensis*	Diptera	Diverse lentic and lotic aquatic habitats	AQUABAC DF3000, (Becker Microbial Products Inc, USA)
			VECTOPRIME (Valent Biosciences, USA)
			TEKNAR (Valent Biosciences, USA)
			VECTOBAC (Valent Biosciences, USA)
			BACTIMOS (Valent Biosciences, USA)
			SOLBAC (Andermatt Biocontrol, Switzerland)
*Lysinibacillus sphaericus*	Diptera: Culicidae	Lentic aquatic habitats	VECTOLEX (Valent Biosciences, USA)
*Serratia entomophila*	Coleoptera: Scarabaeidae	Pastures	BIOSHIELD GRASS GRUB (Biostart, New Zealand)
*Paenibacillus popilliae*	Japanese beetle larvae/grub	Lawns, flowers, mulch beds, gardens	MILKY SPORE POWDER (St. Gabriel Organics, USA)

**Table 2:** Examples of common commercially available entomopathogenic fungi (EPF) and their target insect groups.

Fungi	Target Pest	Crop	Product and Company
*Beauveria bassiana* sensu lato	Psyllids, whiteflies, thrips, aphids, mites	crops	BOTE GHA (Certis, USA)
	Flies, mites, thrips, leafhoppers, and weevils	cotton, glasshouse crops	NATURALIS (Troy Biosciences, USA)
	Coffee berry borer	coffee	CONIDIA (AgroEvo, Germany)
	Whiteflies, aphids, thrips	field crops	MYCOTROL (Bioworks, USA)
	Whiteflies, aphids, thrips	field crops	BOTANIGRAD (Bioworks, USA)
	Corn borer	maize	OSTRINIL (Arysta Lifescience, France)
	Spotted mite, eucalyptus weevil, coffee *borer,* and *whitefly*	crops	BOVERIL (Koppert, The Netherlands)
	Flies		BALANCE (Rincon-Vitova Insectaries, USA)
	As soil treatment	crops	BEAUVERIA BASSIANA PLUS, (BuildASoil, USA)
	Whitefly	peppers, tomatoes, potatoes, eggplants	BEA-SIN (Agrobionsa, Mexico)
*B. brongniartii*	May beetle	forests, vegetables, fruits, grasslands	MELOCONT PILZGERSTE (Samen-schwarzenberger, Austria)
	Cockchafer larvae	Fruits, Meadows	BEAUPRO (Andermatt Biocontrol, Switzerland)
	Scarabs beetle larvae	sugarcane	BETEL (Natural Plant Protection, France)
	Cockchafer	fruits, Meadows	BEAUVERIA-SCHWEIZER (Eric Schweizer, Switzerland)
*Metarhizium anisopliae* sensu lato	Sugar cane root leafhopper	sugarcane	METARRIL WP (Koppert, The Netherlands)
	Cockroaches	houses	BIO-PATH (EcoScience, USA)
	Vine weevils, sciarid flies, wireworms and thrips pupae	glasshouse, ornamental crops	BIO 1020 (Bayer, Germany)
	White grubs	sugarcane	BIOCANE (BASF, Australia)
	termites		BIOBLAST (Paragon, USA)
	Black vine weevil, strawberry root weevil, thrips	stored grains and crops	MET-52 (Novozymes, USA)
	Pepper weevil	chili and bell peppers	META-SIN (Agrobionsa, Mexico)
*M. acridum*	Locusts and grasshoppers	crops	GREEN GUARD (BASF, Australia)
*M. frigidum*	Scarab larvae	crops	BIOGREEN (BASF, Australia)
*M. brunneum*	Wireworms	potato and asparagus crops	ATTRACAP (Biocare, Germany)
*Cordyceps fumosorosea*	Whiteflies	glasshouse crops	PREFERAL WG (Biobest, Belgium)
	Aphids, Citrus psyllid, spider mite, thrips, whitefly	wide range of crops	PFR-97 20% WDG (Certis, USA)
	Whitefly	Peppers, tomatoes, potatoes, eggplants	BEA-SIN (Agrobionsa, Mexico)
	Cotton bullworm, Citrus psyllid	Field crops	CHALLENGER (Koppert, The Netherlands)
*Lecanicillium longisporum*	Aphids	crops	VERTALEC (Koppert, The Netherlands)
	Whiteflies, thrips	crops	MYCOTAL (Koppert, The Netherlands)
*L. lecanii*	Aphids	peppers, tomatoes, potatoes, eggplants	VERTI-SIN (Agrobionsa, Mexico)

**Table 3:** Studies on the isolation of common entomopathogenic fungi from different soil types through insect baiting or soil suspension culture on selective medium.

Entomopathogenic Fungi	Soil Habitat Type	Medium for Soil Suspension Culture	Insect Baiting ^a^	Reference
*Beauveria bassiana* sensu lato	Organically managed farm and hedgerows with hawthorn, poplar, nettles, in Bakkegården, Denmark	n/a	GM	[80]
	Conventional and organic corn field and soybean field; and field margins with grass strips in Iowa, USA	Appendix A, Medium 14 (supplemented with 0.62 gL^−1^ dodine)	GM	[57]
	Agricultural habitat and natural habitat, Southern Ontario and the Kawartha Lakes region, Canada	n/a	GM	[76]
	Cultivated habitats (olive and stone-fruit crops, horticultural crops, cereals crops, leguminous crops, and sunflower); and natural habitats (natural forests, pastures, riverbanks, and desert areas) in Spain and the Canary and the Balearic Archipelagos	n/a	GM	[81]
	Three conventional citrus farms and three organic citrus farms in the Eastern Cape province, South Africa	n/a	*C. capitata*; *T. leucotreta*; GM	[109]
	Cornfields, Iowa, USA	n/a	*D. virgifera virgifera*; TM; GM	[110]
	Tejocote orchard soils, Mexico	n/a	GM	[86]
	Solovakian crop fields, meadows, hedgerows, and forests	Appendix A, Medium 15	GM	[88,97]
	Darmstadt surroundings, Germany	n/a	GM	[73]
	Fields in east, north, central and south west of Switzerland	Appendix A, Medium 15	GM	[61]
	Argan forests in Morocco	Appendix A, Medium 15	GM	[95]
	Natural and cultivated soils, Finland	n/a	*A. aedilis*; *T. castaneum*; GM; TM	[104]
	Native woodland soils, Iceland	n/a	GM; TM	[106]
	Field crop and hedgerows, Årslev, Denmark	n/a	GM	[126]
	Soils from *Dylas* plant community, Greenland	n/a	GM	[107]
	Vineyard soils and hedgerows, Douro wine region, Portugal	n/a	GM; TM	[7]
	Vineyards in the states of New South Wales and Victoria, Australia	Appendix A, Medium 9 (supplemented with 0.2 g/L dodine, 0.1 g/L chloramphenicol, and 0.05 g/L streptomycin sulphate); Appendix A, Medium 15	TM	[127]
*B. brongniartii*	Solovakian crop fields, hedgerows, and forests	n/a	GM	[88]
	Fields in east, north, central, and southwest Switzerland	Appendix A, Medium 15	GM	[61,62]
*B*. *pseudobassiana*	Tejocote orchard soils, Mexico	n/a	GM	[86]
	Solovakian crop fields, meadows, hedgerows, and forests	n/a	GM	[88]
	Hedgerows around an organic farming field, Bakkegården, Denmark	n/a	GM	[128]
	Soils from grasses, *Salix*, and *Betula* community, Greenland	n/a	GM	[107]
	Hedgerows in vineyards, Douro wine region, Portugal	n/a	GM	[7]
	Vineyards in the states of New South Wales and Victoria, Australia	n/a	TM	[127]
*B. australis*	Vineyards in the states of New South Wales and Victoria, Australia	Appendix A, Medium 9 (supplemented with 0.2 g/L dodine, 0.1 g/L chloramphenicol, and 0.05 g/L streptomycin sulphate); Appendix A, Medium 15	TM	[127]
*B*. *varroae*	Hedgerows in vineyards, Douro wine region, Portugal	n/a	GM	[7]
*Clonostachys rosea* f. *rosea*	Vineyard soils and hedgerows, Douro wine region, Portugal	n/a	GM; TM	[7]
*Conidiobolus coronatus*	Organically managed farm in Bakkegården, Denmark	n/a	GM	[80]
	Three conventional citrus farms and three organic citrus farms in the Eastern Cape province, South Africa	n/a	*C. capitata*	[109]
*Cordyceps farinosa*	Organically managed farm; Hedgerows with hawthorn, poplar, nettles in Bakkegården, Denmark	n/a	GM	[80]
	Agricultural habitat and natural habitat, Southern Ontario and the Kawartha Lakes region, Canada	n/a	GM	[76]
	Crop fields, meadows, hedgerows, and forests, Slovakia	n/a	GM	[97]
	Darmstadt surroundings, Germany	n/a	GM	[73]
	Natural and cultivated soils, Finland	n/a	*A. aedilis*; *T. castaneum*; TM	[104]
	Natural soils, Finland	n/a	GM	[104]
	Native woodland soils, Iceland	n/a	GM; TM	[106]
	Field crop and hedgerows, Årslev, Denmark	n/a	GM	[126]
	Soils from grasses and *Salix* community, Greenland	n/a	GM	[107]
*C. fumosorosea*	Organically managed farm and Hedgerows with hawthorn, poplar, nettles in Bakkegården, Denmark	n/a	GM	[80]
	Agricultural habitat and natural habitat, Southern Ontario and the Kawartha Lakes region, Canada	n/a	GM	[76]
	Crop fields, meadows, hedgerows, and forests, Slovakia	Appendix A, Medium 15	GM	[97]
	Darmstadt surroundings, Germany	n/a	GM	[73]
	Fields in east, north, central and south west of Switzerland	Appendix A, Medium 15	GM	[61]
	Cultivated soils, Finland	n/a	*A. aedilis*; *T. castaneum*	[104]
	Natural and cultivated soils, Finland	n/a	TM	[104]
	Natural soils, Finland	n/a	GM	[104]
	Hedgerows, Årslev, Denmark	n/a	GM	[126]
	Soils from *Dyras*, *Salix*, and *Vaccinium* plant communities, Greenland	n/a	GM	[107]
*Lecanicillium* spp.	Organically managed farm in Bakkegården, Denmark	n/a	GM	[80]
	Three conventional citrus farms and three organic citrus farms in the Eastern Cape province, South Africa	n/a	*C. capitata*	[109]
	Vineyard soils, Douro wine region, Portugal	n/a	GM; TM	[7]
*Metarhizium anisopliae* sensu lato and/or *M*. *robertsii*	Organically managed farm in Bakkegården, Denmark	n/a	GM	[80]
	Conventional and organic corn field and soybean field; and field margins with grass strips, Iowa, USA	Appendix A, Medium 14 (supplemented with 0.39 gL^−1^ dodine and 0.25 gL^−1^)	GM	[57]
	Agricultural habitat and natural habitat, Southern Ontario and the Kawartha Lakes region, Canada	n/a	GM	[76]
	Three conventional citrus farms and three organic citrus farms in the Eastern Cape province, South Africa	n/a	*T. leucotreta;* GM	[109]
	Cornfields, Iowa, USA	n/a	*D. virgifera virgifera*; TM; GM	[110]
	Tejocote orchard soils, Mexico	n/a	GM	[86]
	Crop fields, meadows, hedgerows, and forests, Slovakia	Appendix A, Medium 15	GM	[97]
	Darmstadt surroundings, Germany	n/a	GM	[73]
	Fields in east, north, central, and southwest Switzerland	Appendix A, Medium 15	GM	[61]
	Argan forests, Morocco	Appendix A, Medium 15	GM	[95]
	Cultivated soils, Finland	n/a	*A. aedilis*; *T. castaneum*	[104]
	Natural and cultivated soils, Finland	n/a	GM; TM	[104]
	Native woodland soils, Iceland	n/a	TM	[106]
	Field crop and hedgerows, Årslev, Denmark	n/a	GM	[126]
	Soils near ant nests, Tropical forest, Panama	Appendix A, Medium 9 (with and without supplementation of 0.01% (*v/v*) dodine, 0.01% (*v/v*) streptomycinsulphate, and 0.005% (*v/v*) chloramphenicol)	GM; TM	[105]
	Soils from grass, sugarcane and lime grass, Acatlán de Pérez Figueroa, Oaxaca, Mexico	Appendix A, Medium 12, Medium 13	GM	[100]
	Field crop and hedgerows, Årslev, Denmark	n/a	TM	[93]
	Vineyard soils, Douro wine region, Portugal	n/a	GM; TM	[7]
	Vineyards in the states of New South Wales and Victoria, Australia	Appendix A, Medium 9, (supplemented with 0.2 g/L dodine, 0.1 g/L chloramphenicol, and 0.05 g/L streptomycin sulphate); Appendix A, Medium 15	TM	[127]
	Corn, soybean and alfalfa field with different farming treatments (chisel-till, no-till, organic 6-year rotation) in Prince George’s County, Maryland, USA	Appendix A, Medium 10 (with varying strength of CTAB); Appendix A, Medium 15 (with varying strength of dodine)	n/a	[99]
	Cultivated habitats (olive and stone-fruit crops, horticultural crops, cereals crops, leguminous crops, and sunflower); and natural habitats (natural forests, pastures, riverbanks, and desert areas) in Spain and the Canary and the Balearic Archipelagos	n/a	GM	[81]
*M. pingshaense*	Sugar cane leaf, Acatlán de Pérez Figueroa, Oaxaca, Mexico	Appendix A, Medium 12, Medium 13	n/a	[100]
	Vineyards in the states of New South Wales and Victoria, Australia	n/a	TM	[127]
	Soybean (no-till), and corn (chisel-till) farming field in Prince George’s County, Maryland, USA	Appendix A, Medium 10 (with varying strength of CTAB); Appendix A, Medium 15 (with varying strength of dodine)	n/a	[99]
*M. brunneum*	Oilseed rape, Winter wheat and Grass pasture, Eastern Denmark	Appendix A, Medium 13	TM	[96]
	Field crop and hedgerows, Årslev, Denmark	n/a	TM	[93]
	Vineyards in the states of New South Wales and Victoria, Australia	Appendix A, Medium 9 (supplemented with 0.2 g/L dodine, 0.1 g/L chloramphenicol, and 0.05 g/L streptomycin sulphate); Appendix A, Medium 15	TM	[127]
	Corn (two systems: organic 6 year rotation; and no-till), and soybean (organic 6 year rotation) farming in Prince George’s County, Maryland, USA	Appendix A, Medium 10 (with varying strength of CTAB); Appendix A, Medium 15 (with varying strength of dodine)	n/a	[99]
*M. guizhouense*	Lime grass soil, Acatlán de Pérez Figueroa, Oaxaca, Mexico	n/a	GM	[100]
	Vineyard soils, Douro wine region, Portugal	n/a	GM	[7]
	Vineyards in the states of New South Wales and Victoria, Australia	n/a	TM	[127]
*M. flavoviride*	Organically managed farm and Hedgerows with hawthorn, poplar, nettles in Bakkegården, Denmark	n/a	GM	[80]
	Three conventional citrus farms and three organic citrus farms in the Eastern Cape Province, South Africa	n/a	*T. leucotreta;* GM	[109]
	Oilseed rape, Winter wheat and Grass pasture, Eastern Denmark	Appendix A, Medium 13	TM	[96]
	Field crop and hedgerows, Årslev, Denmark	n/a	TM	[93]
	Vineyards in the states of New South Wales and Victoria, Australia	Appendix A, Medium 9 (supplemented with 0.2 g/L dodine, 0.1 g/L chloramphenicol, and 0.05 g/L streptomycin sulphate); Appendix A, Medium 15	TM	[127]
*M. majus*	Grass pasture, Eastern Denmark	Appendix A, Medium 13	n/a	[96]
	Vineyards in the states of New South Wales and Victoria, Australia	Appendix A, Medium 9 (supplemented with 0.2 g/L dodine, 0.1 g/L chloramphenicol, and 0.05 g/L streptomycin sulphate); Appendix A, Medium 15	n/a	[127]
*Purpureocillium lilacinum*	Argan forests in Morocco	Appendix A, Medium 15	GM	[95]
	Vineyard soils, Douro wine region, Portugal	n/a	GM; TM	[7]

^a^ Bait insects *G. mellonella* and *T. molitor* are abbreviated as GM and TM, respectively.

## Data Availability

Not applicable.

## Appendix A

Common culture medium used for the isolation of entomopathogenic bacteria.

(1) **Caprylate-thallous agar (CTA).**

This medium is made by mixing two solutions, i.e., A and B. Both these medium should be autoclaved separately and added aseptically.

(1a) Solution A

**Reagents and Chemicals**	**Chemical Formula (If Applicable)**	**Quantity**
Monopotassium phosphate	KH_2_PO_4_	0.68 g
Magnesium sulfate heptahydrate	MgSO_4_.7H_2_O	0.3 g
Dipotassium phosphate	K_2_HPO_4_	0.15 g
Thallium(I) sulphate	Tl_2_SO_4_	0.25 g
Yeast Extract		1 g
Calcium chloride	CaCl_2_	0.1 g
Caprylic (n-octanoic) acid	CH_3_(CH_2_)_6_.COOH	1.1 mL
Trace element solution		10 mL
Distilled water	H_2_O	1 L

Note: Thallium (I) sulphate is extremely toxic so it should be used with caution. The pH should be adjusted to 7.2 either by increasing it using K_2_HPO_4_ or decreasing it is using KH_2_PO_4_.

Trace element solution

**Reagents and Chemicals**	**Chemical Formula (If Applicable)**	**Quantity**
Ferrous sulphate heptahydrate	FeSO_4_.7H_2_O	0.055 g
Trihydrogen phosphate	H_3_PO_4_	1.96 g
Zinc sulphate heptahydrate	ZnSO_4_.7H_2_O	0.0287 g
Manganese(II) sulphate monohydrate	MnSO_4_.H_2_O	0.0223 g
Copper(II) sulphate pentahydrate	CuSO_4_.5H_2_O	0.0025 g
Cobalt(II) nitrate hexahydrate	Co(NO_3_)_2_.6H_2_O	0.003 g
Boric acid	H_3_BO_3_	0.0062 g
Distilled water	H_2_O	1 L

Note: Once made the trace element solution can be kept for months at 4 °C.

(1b) Solution B

**Reagents and Chemicals**	**Chemical Formula (If Applicable)**	**Quantity**
Ammonium sulphate	(NH_4_)_2_SO_4_	1.0 g
Sodium chloride	NaCl	7.0 g
Agar		15 g
Distilled water	H_2_O	1 L

(2) **Deoxyribonuclease (DNase)-Toluidine Blue agar.**

**Reagents and Chemicals**	**Chemical Formula (If Applicable)**	**Quantity**
Deoxyribonuclease test agar		37.8 g
Toluidine blue 0.1% *w/v* solution	NaCl	90.0 ml
L-arabinose	C_5_H_10_O_5_	10.0 g
Distilled water	H_2_O	900 mL

(3) **St. Julian medium (J-medium).**

**Reagents and Chemicals**	**Chemical Formula (If Applicable)**	**Quantity**
Yeast extract		15 g
Tryptone		5 g
Dipotassium phosphate	K_2_HPO_4_	3 g
Glucose (sterilized by filtration)	C_6_H_12_O_6_	2.0 g
Distilled water	H_2_O	1 L

Note: Adjust the pH to 7.3–7.5 and autoclave. For plate culture, add 20 g agar. Add glucose after autoclaving.

(4) **Mueller-Hinton broth, yeast extract, potassium phosphate, glucose and pyruvate (MYPGP) medium.**

**Reagents and Chemicals**	**Chemical Formula (If Applicable)**	**Quantity**
Dipotassium phosphate	K_2_HPO_4_	3.0 g
Sodium pyruvate	C_3_H_3_O_3_Na	1.0 g
Mueller-Hinton broth		10.0 g
Glucose (sterilized by filtration)	C_6_H_12_O_6_	2.0 g
Yeast Extract		10.0 g
Distilled water		1 L

Note: Adjust the pH to 7.1 and autoclave. For plate culture, add 20 g agar. Add glucose after autoclaving.

(5) **Adonitol agar.**

**Reagents and Chemicals**	**Chemical Formula (If Applicable)**	**Quantity**
Sodium chloride	NaCl	4.17 g
Adonitol	C_5_H_12_O_5_	5.0 g
Peptone		8.33 g
Bacto agar		12.5 g
Bromothymol blue solution	C_27_H_28_Br_2_O_5_S	10 mL
Distilled water	H_2_O	990 mL

Note: Adjust the pH to 7.4 before adding bromothymol blue solution.

Bromothymol blue solution

**Reagents and Chemicals**	**Chemical Formula (If Applicable)**	**Quantity**
Bromothymol blue	C_27_H_28_Br_2_O_5_S	0.2 g
Sodium hydroxide (0.1M)	NaOH	5 mL
Distilled water	H_2_O	900 mL

(6) **Itaconate agar.**

**Reagents and Chemicals**	**Chemical Formula (If Applicable)**	**Quantity**
Monopotassium phosphate	KH_2_PO_4_	3.0 g
Disodium phosphate	Na_2_HPO_4_	6.0 g
Sodium chloride	NaCl	0.5 g
Ammonium chloride	NH_4_Cl	1.0 g
Calcium chloride solution (sterilised) (0.01M)	CaCl_2_	10.0 mL
Magnesium sulfate heptahydrate (sterilised) (1M)	MgSO_4_.7H_2_O	1.0 mL
Itaconic acid solution (filter sterilised) (20%)	C_5_H_6_O_4_	10 mL
Distilled water	H_2_O	1 L

Note: Adjust the pH to 7.0 before autoclaving.

(7) **Minimal Basal Salt (MBS) medium.**

**Reagents and Chemicals**	**Chemical Formula (If Applicable)**	**Quantity**
Monopotassium phosphate	KH_2_PO_4_	6.8 g
Magnesium sulfate heptahydrate	MgSO_4_.7H_2_O	0.3 g
Manganese monohydrate sulphate	MnSO_4_.1H_2_O	0.02 g
Ferric sulfate	Fe_2_(SO_4_)_3_	0.02 g
Zinc sulfate heptahydrate	ZnSO_4_.7H_2_O	0.02 g
Calcium chloride	CaCl_2_	0.2 g
Tryptone		10 g
Yeast Extract		2 g

Note: Adjust the pH to 7.2 before autoclaving.

Common culture medium used for the isolation of entomopathogenic fungi.

(8) **Potato Dextrose agar (PDA)**

**Reagents and Chemicals**	**Chemical formula (If Applicable)**	**Quantity**
Potato dextrose agar		39.0 g
Distilled water	H_2_O	1 L

(9) **Sabouraud Dextrose agar (SDA)**

**Reagents and Chemicals**	**Chemical Formula (if Applicable)**	**Quantity**
Sabouraud dextrose agar		65.0 g
Distilled water	H_2_O	1 L

(10) **Oatmeal Cetyl Trimethyl Ammonium Bromide (OM-CTAB) agar.**

**Reagents and Chemicals**	**Chemical Formula (If Applicable)**	**Quantity**
Oatmeal (cooked in distilled water)		20.0 g
Cetyl trimethyl ammonium bromide (CTAB)	C_19_H_42_BrN	0.6 g
Chloramphenicol	C_11_H_12_Cl_2_N_2_O_5_	0.5 g
Agar		20 g
Distilled water	H_2_O	To make upto 1L

(11) **Dichloran Rose-Bengal Chloramphenicol agar (DRBCA).**

This medium is easily available as powder and sold by the majority of the culture media suppliers.

**Reagents and Chemicals**	**Chemical Formula (If Applicable)**	**Quantity**
Dichloran Rose-Bengal Chloramphenicol agar		32.0 g
Distilled water	H_2_O	1 L

(12) ***Metarhizium* Medium**

**Reagents and Chemicals**	**Chemical Formula (If Applicable)**	**Quantity**
Glucose	C_6_H_12_O_6_	10.0 g
Peptone		10.0 g
Oxgall		15.0 g
Agar		35.0 g
Dodine (N-dodecylguanidine monoacetate)	C_15_H_33_N_3_O_2_	10 mg
Cycloheximide	C_15_H_23_NO_4_	250 mg
Chloramphenicol	C_11_H_12_Cl_2_N_2_O_5_	500 mg
Distilled water	H_2_O	1 L

Note: Cyclohexamide is quite toxic and caution is needed while handling.

(13) **Chloramphenicol Thiabendazole Cycloheximide (CTC) medium.**

**Reagents and Chemicals**	**Chemical Formula (If Applicable)**	**Quantity**
Potato dextrose agar		39.0 g
Yeast extract		0.5 g
Chloramphenicol	C_11_H_12_Cl_2_N_2_O_5_	500 mg
Thiabendazole	C_10_H_7_N_3_S	1 mg
Cycloheximide	C_15_H_23_NO_4_	250 mg
Distilled water	H_2_O	1 L

(14) **Oatmeal Dodine agar (ODA).**

**Reagents and Chemicals**	**Chemical Formula (If Applicable)**	**Quantity**
Oatmeal infusion		20.0 g
Dodine (N-dodecylguanidine monoacetate)	C_15_H_33_N_3_O_2_	550 mg
Chlortetracycline	C_22_H_23_ClN_2_O_8_	5 mg
Crystal violet	C_25_N_3_H_30_Cl	10 mg
Agar		20.0 g
Distilled water	H_2_O	1 L

(15) **Sabouraud-2-Glucose agar (S2GA).**

**Reagents and Chemicals**	**Chemical Formula (If Applicable)**	**Quantity**
Glucose	C_6_H_12_O_6_	20.0 g
Peptone		10.0 g
Streptomycin sulphate	C_42_H_84_N_14_O_36_S_3_	600 mg
Tetracycline	C_22_H_24_N_2_O_8_	50 mg
Cycloheximide	C_15_H_23_NO_4_	50 mg
Dodine (N-dodecylguanidine monoacetate)	C_15_H_33_N_3_O_2_	100 mg
Agar		12.0 g
Distilled water	H_2_O	1 L

(16) ***Purpureocillium lilacinum* medium.**

**Reagents and Chemicals**	**Chemical formula (If Applicable)**	**Quantity**
Potato dextrose agar		39.0 g
Sodium chloride	NaCl	10–30 g
Tergitol		1 g
Pentachloronitrobenzene	C_6_Cl_5_NO_2_	500 mg
Benomyl	C_14_H_18_N_4_O_3_	500 mg
Streptomycin sulphate	C_42_H_84_N_14_O_36_S_3_	100 mg
Chlortetracycline hydrochloride	C_22_H_24_C_l2_N_2_O_8_	50 mg
Distilled water	H_2_O	1 L

(17) ***Lecanicillium*-specific medium.**

**Reagents and Chemicals**	**Chemical Formula (If Applicable)**	**Quantity**
L-sorbose	C_6_H_12_O_6_	2 g
L-asparagine	C_4_H_8_N_2_O_3_	2 g
Dipotassium phosphate	K_2_HPO_4_	1 g
Potassium chloride	KCl	1 g
Magnesium sulfate heptahydrate	MgSO_4_.7H_2_O	0.5 g
Ferric-sodium salt (FeNaEDTA)	C_10_H_12_N_2_O_8_FeNa	0.01 g
Agar		20 g
Streptomycin sulphate	C_42_H_84_N_14_O_36_S_3_	0.3 g
Chlortetracycline hydrochloride	C_22_H_24_C_l2_N_2_O_8_	0.05 g
Pentachloronitrobenzene	C_6_Cl_5_NO_2_	0.8 g
Borax	NaB_4_O_7_.10H_2_O	1 g
Distilled water		1 L

Note: Adjust the pH to 4.0 using 10% trihydrogen phosphate (H_3_PO_4_) before autoclaving.
